# Human Migration through Bottlenecks from Southeast Asia into East Asia during Last Glacial Maximum Revealed by Y Chromosomes

**DOI:** 10.1371/journal.pone.0024282

**Published:** 2011-08-31

**Authors:** Xiaoyun Cai, Zhendong Qin, Bo Wen, Shuhua Xu, Yi Wang, Yan Lu, Lanhai Wei, Chuanchao Wang, Shilin Li, Xingqiu Huang, Li Jin, Hui Li, the Genographic Consortium

**Affiliations:** 1 Ministry of Education Key Laboratory of Contemporary Anthropology, School of Life Sciences and Institutes of Biomedical Sciences, Fudan University, Shanghai, China; 2 Chinese Academy of Sciences and Max Planck Society Partner Institute for Computational Biology, Shanghai Institutes for Biological Sciences, Chinese Academy of Sciences, Shanghai, China; 3 Institute of Ethnology and Anthropology, Guangxi University for Nationalities, Nanning, Guangxi, China; 4 Institute of Health Sciences, China Medical City, Taizhou, Jiangsu, China; University of Utah, United States of America

## Abstract

Molecular anthropological studies of the populations in and around East Asia have resulted in the discovery that most of the Y-chromosome lineages of East Asians came from Southeast Asia. However, very few Southeast Asian populations had been investigated, and therefore, little was known about the purported migrations from Southeast Asia into East Asia and their roles in shaping the genetic structure of East Asian populations. Here, we present the Y-chromosome data from 1,652 individuals belonging to 47 Mon-Khmer (MK) and Hmong-Mien (HM) speaking populations that are distributed primarily across Southeast Asia and extend into East Asia. Haplogroup O3a3b-M7, which appears mainly in MK and HM, indicates a strong tie between the two groups. The short tandem repeat network of O3a3b-M7 displayed a hierarchical expansion structure (annual ring shape), with MK haplotypes being located at the original point, and the HM and the Tibeto-Burman haplotypes distributed further away from core of the network. Moreover, the East Asian dominant haplogroup O3a3c1-M117 shows a network structure similar to that of O3a3b-M7. These patterns indicate an early unidirectional diffusion from Southeast Asia into East Asia, which might have resulted from the genetic drift of East Asian ancestors carrying these two haplogroups through many small bottle-necks formed by the complicated landscape between Southeast Asia and East Asia. The ages of O3a3b-M7 and O3a3c1-M117 were estimated to be approximately 19 thousand years, followed by the emergence of the ancestors of HM lineages out of MK and the unidirectional northward migrations into East Asia.

## Introduction

The origin of early East Asians, and particularly their point of entry into the region, remains ambiguous [1]. Fortunately, the phylogeographic power of the Y chromosome for reconstructing human population history [2] has been effectively used to address the question of the origin of East Asians. Some have hypothesized that haplogroups C and D derived from the African exodus conducted the first northward population movement of modern humans to East Asia and became the first successful modern human colonizers of that region [3]. Previous studies have shown that the most prevalent haplogroup O-M175 accounts for at least 57% of the Y chromosomes in East Asia, standing out as the most relevant material to address the question of the origin of East Asians [4]. These studies also indicated that the three downstream haplogroups derived from O-M175 (O3-M122, O2a-M95, and O1a-M119) entered East Asia from the south [4]–[8], suggesting the importance of the southern entrance for East Asian ancestors migrating from Southeast Asia. The study on Hainan aborigines proved that only O1a-M119 and O2a-M95 has gone through the coastal southern entrance in the east, and there must be other entrance(s) for O3-M122 [9]. By studying more population samples and undertaking more detailed analyses of Y chromosome haplogroup subdivisions in populations at juncture of Southeast and East Asia, we might be able to generate, stage by stage, a more detailed history of the emergence of East Asians out of Southeast Asians.

**Figure 1:**
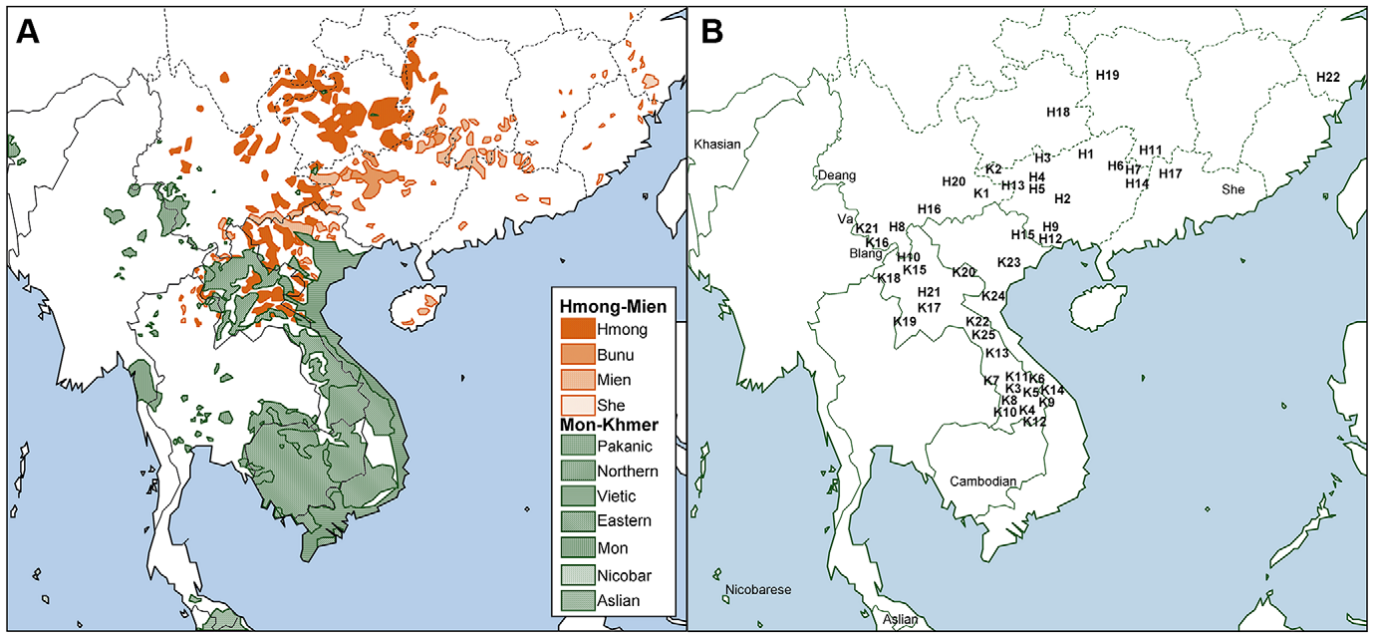
Distribution of Hmong-Mien and Mon-Khmer populations. A: distribution of the two ethnic groups; B: our population samples.

Mon-Khmer (MK) and Hmong-Mien (HM) speaking populations live in the area where the expansion towards East Asia from Southeast Asia is thought to have occurred (Fig. 1A). Despite this fact, very few studies of the genetic structure of these populations have been conducted. The MK linguistic phylum contains 147 languages, and their speakers exceed 90 million, scattering in the Indo-China peninsula from Assam to Vietnam. The HM linguistic phylum, spoken in South China and Southeast Asia, comprises 38 languages, with a population over 12 million in China (2000 Census), and more than one million outside of China [10]. There are also large populations of Sino-Tibetan (ST) and Tai-Kadai (TK) populations in the Indo-China Peninsula, but they are probably recent migrants from the northern and eastern parts of East Asia, respectively, as no record of their presence in this region prior to 3,000 years ago has been found [6]–[8], [11]. Only MK and HM populations are aboriginal to the juncture region of East Asia and Southeast Asia, and therefore they are essential in revealing the process of modern humans' entrance into East Asia.

In addition, among the three derived haplogroups, O2a-M95 and O3-M122 are the most abundant in Southeast Asia. However, M95 (originated around 65,000 years before present, hereafter YBP, 95% C.I. 25,442–132,230YBP [12]) and M122 (originated about 18,000–60000 YBP [5],at least 30,000 YBP [4]) are both very old and have accumulated too much diversity and phylogenetic complexity to be useful for tracing the migratory history from Southeast Asia to East Asia compared to relatively younger haplogroups. High diversity, thereby large ‘effective population size’ of certain haplogroup could have weakened signal of any possible genetic drift the populations might have undergone. In this paper, after we analyzed all the haplogroups found in the MK and HM populations, we focused on O3a3b-M7 and O3a3c1-M117, two sub-haplogroups of O3-M122, whose age matched with the time (around 20,000 YBP) for early human migrated from Southeast Asia into East Asia [4], [13]. In addition, both haplogroups occurs at high frequencies in our samples of MK and HM. Moreover, the O3a3b haplogroup is almost absent in other population groups. Moreover, the short tandem repeat (STR) diversity was highly informative with respect to reconstructing migratory pathways in the area of interest.

## Materials and Methods

### Population Samples

Blood samples of 1,652 unrelated male individuals from 22 HM populations and 25 MK populations were collected (Table 1 and Fig. 1B) from unrelated healthy males. The donors gave informed consent to participate in the study, and signed the consent form in their languages. The ethics committee for biological researches at Fudan School of Life Sciences approved the study.

**Table 1:** Frequencies of Y chromosome SNP haplogroups in Hmong-Mien and Mon-Khmer populations.

					Haplogroups and diagnostic SNPs																
No.^a^	Phyla^b^	Population	ISO639-3	Size	C	DE*	D1	F*	K*	O*	O3*	O3a3b	O3a3c*	O3a3c1	O1a*	O1a2	O2*	O2a*	O2a1	Q1a1	P*
					M130	YAP	M15	M89	M9	M175	M122	M7	M134	M117	M119	M110	P31	M95	M88	M120	M45
H1	HM-N	Pahng^c^	PHA	31					19.35	32.26	12.90	**3.20**	6.50	**12.90**			nd	**12.90**			
H2	HM-N	Bunu, Southern	BWX	20	5.00					20.00	15.00	**10.00**			20.00		nd	**10.00**	20.00		
H3	HM-N	Bunu, Northern^c^	BWX	10	50.00					20.00		**30.00**					nd				
H4	HM-N	Bunu, Woodhandle	BWX	6	50.00		16.67				16.67								16.67		
H5	HM-M	Kimmun,Blue	MJI	28				3.57		3.57		**3.57**	39.29	**32.14**			nd	**17.86**			
H6	HM-M	Yao, Lowland	MJI	31	3.23		19.35	3.23			12.90			**16.13**				**16.13**	29.03		
H7	HM-M	Kimmun, Lowland	MJI	41	2.44		7.32	2.44	4.88	4.88	14.63		7.32	**12.20**	12.20		nd	**24.39**	7.32		
H8	HM-M	Kimmun, Mountain	MJI	32	3.13					3.13	6.25		50.00	**6.25**			nd	**31.25**			
H9	HM-M	Mien, Southern	IUM	31					9.68	32.26	6.45	**6.45**		**6.45**	6.45		nd	**19.35**	12.90		
H10	HM-M	Mien, Top board	IUM	11	9.09		9.09	9.09			9.09	**9.09**		**27.27**	9.09			**9.09**	9.09		
H11	HM-M	Mien,Mountain Straggler	IUM	20					10.00		25.00	**5.00**	10.00	**35.00**	10.00				5.00		
H12	HM-M	Mien, Flower-head	IUM	19						21.05	15.79	**5.26**		**31.58**			nd	**21.05**	5.26		
H13	HM-M	Mien, Northern	IUM	33	3.03					9.09	21.21		12.12	**18.18**	9.09		nd	**21.21**	6.06		
H14	HM-M	Mien, Native	IUM	41			12.20				58.54	**7.32**	4.88	**7.32**				**7.32**	2.44		
H15	HM-M	Mien, Thin board	IUM	11				9.09		18.18	9.09			**27.27**	18.18		nd	**9.09**	9.09		
H16	HM-M	Mien, Western^c^	IUM	47			4.30		8.51	14.89	23.40	**2.10**	4.30	**23.40**	4.30		nd	**14.90**			
H17	HM-M	Zaomin	BPN	37	5.41				2.70	5.41	16.22	**32.43**	2.70	**13.51**			nd		21.62		
H18	HM-H	Miao,Guizhou^c^	HMQ	49	8.16		2.04		4.08	24.49	24.49	**4.08**		**2.04**	12.24		nd	**16.33**	2.04		
H19	HM-H	Miao,Hunan	MMR	100	14.00		1.00	2.00	9.00	9.00	24.00	**4.00**	7.00	**9.00**	7.00		nd	**9.00**	5.00		
H20	HM-H	Miao,Yunnan^c^	HMD	49	6.12		6.12		2.04	12.24	18.37	**12.24**		**6.12**	6.12		nd	**30.61**			
H21	HM-H	Hmong,Daw	MWW	51	25.49		7.84		5.88	3.92		**33.33**		**7.84**	1.96		nd	**5.88**	5.88	1.96	
H22	HM-S	She, Northern	SHX	56	3.57			1.79		3.57	30.36	**25.00**	16.07	**7.14**	8.93		nd	**3.57**			
K1	MK-B	Bugan	BBH	32					3.13	31.25			6.25	**9.38**			nd	**50.00**			
K2	MK-B	Palyu	PLY	30			3.33		3.33	10.00	30.00		6.67	**10.00**	10.00	3.33	nd	**23.33**			
K3	MK-E	Alak	ALK	31						6.67		**13.33**		**3.33**				**56.67**	16.67		3.33
K4	MK-E	Brau	BRB	32				3.13			3.13	**25.00**					3.13	**62.50**	3.13		
K5	MK-E	Inh	IRR	34						2.94	2.94	**8.82**		**2.94**			2.94	**79.41**			
K6	MK-E	Jeh	JEH	32						6.25		**46.88**						**46.88**			
K7	MK-E	Suy	KDT	39						5.13	5.13	**2.56**						**56.41**	30.77		
K8	MK-E	Kataang	KGD	38							21.62	**16.22**		**16.22**			5.41	**10.81**	27.03		2.70
K9	MK-E	Katu	KUF	45						2.22		**22.22**			6.67			**68.89**			
K10	MK-E	Laven	LBO	50	2.00	24.00		4.00	4.00	4.00	2.00	**12.00**		**2.00**	2.00			**42.00**	2.00		
K11	MK-E	Ngeq	NGT	35								**48.57**						**48.57**			2.86
K12	MK-E	Oy	OYB	50								**34.00**		**2.00**			2.00	**60.00**	2.00		
K13	MK-E	So	SSS	50					12.00	6.00	6.00	**8.00**		**2.00**			12.00	**42.00**	12.00		
K14	MK-E	Talieng	TDF	35	2.86					2.86		**22.86**		**2.86**			2.86	**62.86**	2.86		
K15	MK-N	Bit	BGK	28					3.57				10.71	**32.14**				**53.57**			
K16	MK-N	Blang^c^	BLR	52	15.38				5.77	9.62	21.15		5.77	**11.54**			nd	**30.77**			
K17	MK-N	Khmu	KJG	51	5.88				3.92		3.92	**1.96**		**13.73**			3.92	**60.78**	5.88		
K18	MK-N	Lamet	LBN	35	5.71						2.86	**5.71**						**85.71**			
K19	MK-N	Mal	MLF	50							4.00	**2.00**	2.00	**14.00**			4.00	**66.00**	8.00		
K20	MK-N	Xinhmul	PUO	29			3.45							**6.90**			3.45	**17.24**	68.97		
K21	MK-N	Ava^c^	WBM	29	6.90				6.90	10.34	44.83		3.45	**27.59**			nd				
K22	MK-V	Bo	BGL	28						7.14	3.57	**7.14**			3.57		7.14	**64.29**	3.57		3.57
K23	MK-V	Kinh^c^	VIE	15				6.67		6.67	33.33		6.67	**6.67**	6.67		nd	**33.33**			
K24	MK-V	Muong^c^	MTQ	12				8.33		8.33	25.00		8.33	**8.33**			nd	**41.67**			
K25	MK-V	Aheu	THM	38				2.63		5.26	15.79						8.90	**52.63**	15.79		

All of these population samples are reported for the first time in this paper except for two populations, H3 from reference [5] and K2 from [31].

a The population sample numbers are the same of those in Figure 1B.

b Phylum abbreviations are as follows: HM-N (Hmong-Mien, Bunu); HM-M (Hmong-Mien, Mien); HM-H (Hmong-Mien, Hmong); HM-S (Hmong-Mien, She); MK-B (Mon-Khmer, Pakanic); MK-E (Mon-Khmer, Eastern Khmer); MK-N (Mon-Khmer, Northern Khmer); MK-V (Mon-Khmer, Vietic).

c Y-STR data of several populations are unavailable due to poor DNA quality or lack of data from references. nd = no data.

Our population samples originated across most of the conjunctive area of Southeast and East Asia. Individuals from the same population were sampled from various locations to ensure adequate coverage. Although the sample sizes of some really small populations were correspondingly small (such as Woodhandle Bunu), they were still useful by pooling them in the analyses.

We collected fingertip blood samples and kept the samples dry on the filter paper. Whole genome amplification was performed with the filter paper samples using the published method [14].

### Genetic markers

Thirty Y chromosome biallelic loci were screened according to the population specificity of East and Southeast Asia. Genotyping of DE*-YAP was conducted by agarose gel electrophoresis directly after PCR. O*-M175, O3a1-M121, O3a3c-M134, O3a3c1-M117, and D1-M15 were scanned on the ABI 3130×l genetic analyzer after Y chromosome PCR with fluorescent primers (Applied Biosystems, Foster City, CA). C-M130, F*-M89, K*-M9, O3*-M122, O3a3b-M7, O3a2-M164, O3a3a-M159, O1a*-M119, O1a1a-M101, O1a2-M110, O2a*-M95, O2a1-M88, Q1a1-M120, P*-M45, and D3-P47 were typed using a PCR-RFLP assay (Protocol S1) [15]. D-M174, D2-M55, NO-M214, N1-LLY22g, N1a-M128, N1b-P43, N1c-M46, P31-O2, and O3a-M324 were typed using TaqMan® SNP genotyping assays.

Seventeen Y chromosome microsatellites were also typed for further study on the diversity of samples. These include DYS456, DYS389I, DYS389II, DYS390, DYS458, DYS19, DYS385I, DYS385II, DYS393, DYS391, DYS439, DYS635, DYS392, YGATAH4, DYS437, DYS438, and DYS448. Multiple-touchdown PCR was adopted (Protocol S1). The scan of the PCR product was also conducted on the ABI 3130×l genetic analyzer, and the software GeneMapper was used to analyze genotype data. Each Y chromosome haplotype was determined on the basis of experimental results according to Y-DNA Haplogroup Tree [16].

### Statistic methods

The frequency contour maps for haplogroups were constructed by Golden Software Surfer7.0. Clustering analyses were performed based on the haplogroup frequency data of various East Asian populations, including principal component analysis and neighbor-joining hierarchical cluster analysis, using SPSS 15.0.

**Figure 2:**
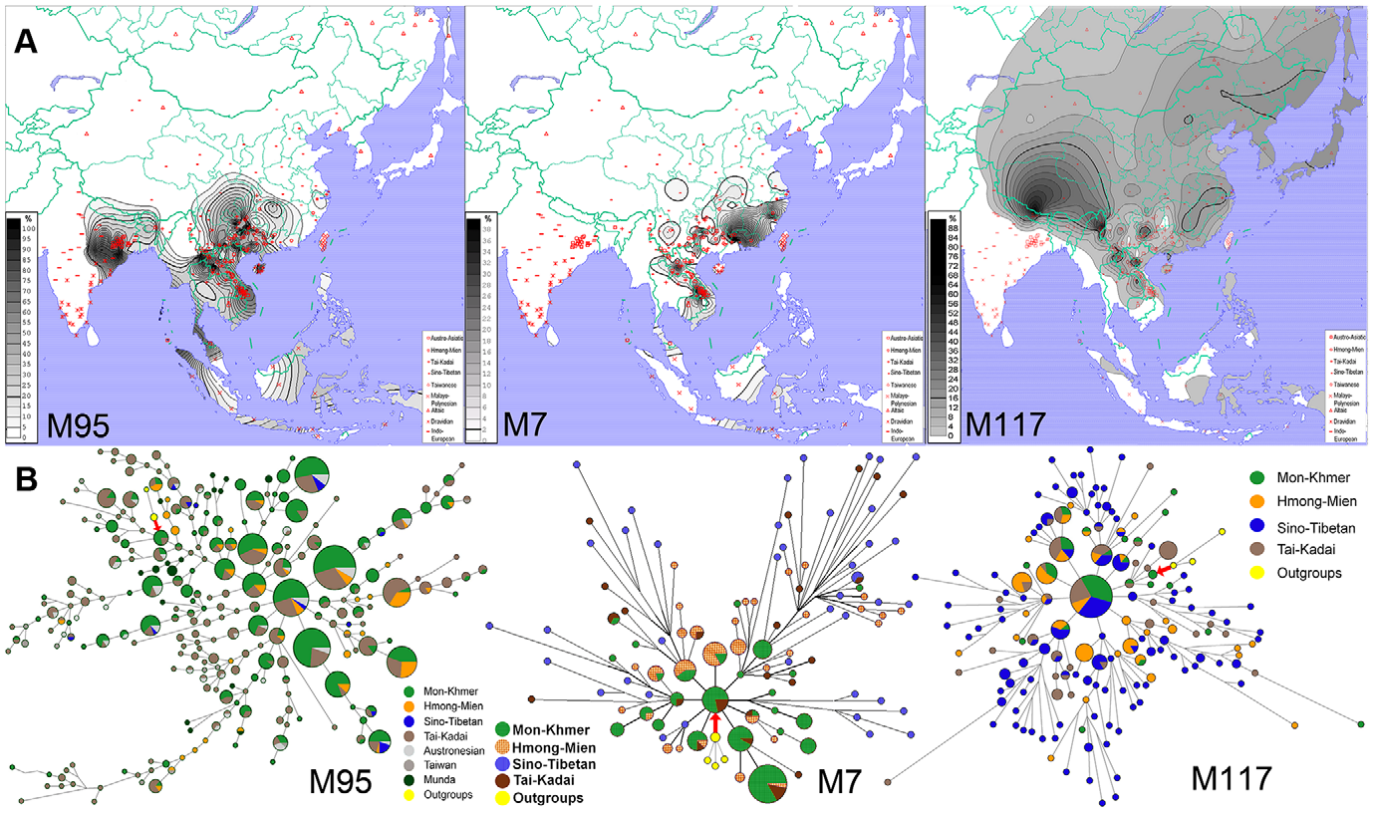
Frequencies and diversities of O2-M95, O3-M7, and O3-M117. A: Geographic distributions of haplogroup frequency; B: STR haplotype Networks.

The networks of Y-STR haplotypes of the individual samples with haplogroups O2a-M95, O3a3b-M7, and O3a3c1-M117 (Fig. 2) were constructed using the NETWORK 4510 program [17]. Only DYS19, DYS389I, DYS389II, DYS390, DYS391, DYS392, and DYS393 were included in the Network analyses of O3a3b-M7 and O3a3c1-M117, and only DYS19, DYS389I, DYS390, DYS391, and DYS393 were included in those of O2a-M95, as the reference data did not include other STR markers [4], [7], [18]. We also constructed the networks of C-M130, D-M174, F*-M89, K*-M9, N1-LLY22g, P-M45, O*-M175, O1a*-M119, O2*-P31, O2a1-M88, O3*-M122, O3a*-M324, and O3a3c*-M134 (Figure S1). Because the fragment size of DYS389II contains those of DYS389I, the genotype of DYS389II was obtained by subtracting the genotype DYS389I. Networks were calculated by the median-joining method (ε = 0) [19], weighting the STR loci according to the average of their relative variability within the haplogroup subclades and after having processed the data with the reduced-median method. The weights assigned took into account the Y-STR variation across the haplogroup: variance 0.0–0.2, weight 10; variance 0.2–0.4, weight 8; variance 0.4–0.6, weight 6; variance 0.6–0.8, weight 4; variance >0.8, weight 2. The root of the network represents individual samples with upstream haplogroups. The structure of the network did not change whether the root was introduced or not.

Based on the Y-STR diversity of the haplogroups O2a, O3a3b, and O3a3c1, the time depth of haplogroups within Network was estimated from the ρ statistic (the mean number of mutations from the assumed root of the network), using a 25-year generation time and a mean per-locus, per-generation mutation rate of 6.9×10^−4^ [20], [21]. We also introduce the average squared difference (ASD) of several STR markers of haplotypes sampled from the present-day population of the haplogroups O2a-M95, O3a3b-M7, and O3a3c1-M117 to estimate the time back to most recent common ancestor (TMRCA) for haplotypes within this lineage, still using the mutation rate of 6.9×10^−4^ per locus per 25 years [21]–[23]. The estimator is
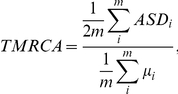
where ASD*i* is the average squared difference in the number of repeats for the *i*th STR between pairs of alleles sampled within haplogroup; *m* is the number of STRs investigated, *µi* is the mutation rate for *i*th STR.

**Figure 3:**
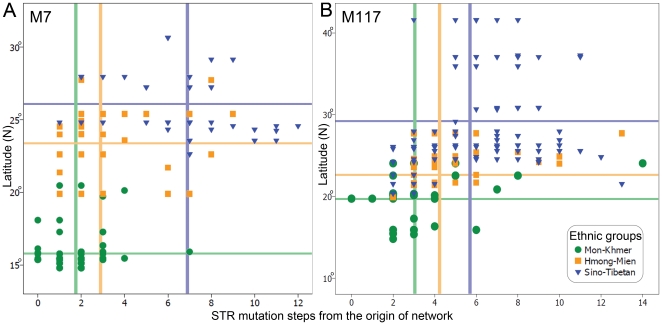
Relationship between the geographic locations of the STR haplotypes and STR mutation steps from the origins of the networks. A: O3-M7; B: O3-M117. X-coordinate represents the STR mutation steps counted from the Network origins, while Y-coordinate represents the latitude of each population with certain haplotypes. The correlations between latitude and mutation step were significant for both M7 (r = 0.551, P = 2.18×10^−17^), and M117 (r = 0.442, P = 4.07×10^−14^).

We analyzed the correlation between the geographic locations and the ages of the STR haplotype samples, to address the origin and diffusion of M7 and M117. The age of a haplotype is mostly related to its location in certain Y-STR network when the network includes sufficient samples, e.g., the haplotypes closer to the center of the network are most probably older. Therefore, in the analysis, we did not use the absolute ages, but the mutation step numbers counted from the center of the network. Coordinate planes were then set up with the mutation steps as x-coordinate and the latitude of each sample as y-coordinate (Fig. 3). The Pearson correlation was judged in the coordinate planes and the significance test of correlation was performed using SPSS 15.0.

To show the result in more animate way, we turned the coordinate planes into two flashes using Adobe ImageReady CS2. In these flashes, the samples occurred on the maps in turn of the ages (Information S1 and Information S2).

## Results

### Y chromosome SNP haplogroups distribution

The haplogroup frequencies of the HM and MK populations are listed in Table 1. The frequencies of haplogroups O3a3b, O3a3c1, and O2a are high in our MK and HM population samples. O2a-M95 (including O2a* and O2a1) is the most frequent haplogroup in MK (87.18%) and HM (45.16%) populations. It is scattered in the area from Northeast India to Southwest China and island Southeast Asia, appeared with high frequencies in different ethnic phyla. This haplogroup has the highest frequencies in Austro-Asiatic (both Munda and Mon-Khmer subfamilies) and Hmong-Mien populations, while it was widely found in Sino-Tibetan, Tai-Kadai, and Austronesian populations, with no clear ethnic association but geographic restriction [4], [12], [24], [25]. The distribution of haplogroup O3a3b-M7 is much more fragmented geographically but is largely restricted to the HM and MK samples (Fig. 2A), while nearly absent in TK, Han Chinese, and other ethnic groups [4]–[8], [24], [26]–[29]. Haplogroup O3a3c1-M117 is found in most of the populations in East and Southeast Asia, and has highest frequency and largest diversity in ST populations. In the distribution map, Tibet, Nepal, and those regions along the entrance of East Asia share high levels of haplogroup O3a3c1-M117 frequency [4], [9], [15], [28], [30] (Fig. 2A). Given the fact that O2a-M95, O3a3b-M7, and O3a3c1-M117 are abundant and shared by MK and HM, they may represent a unique genetic tool for studying population migration in the area connecting Southeast and East Asia.

History of the large populations such as East Asians was always complicated. The population was formed by many demographic evens, represented by different Y chromosome haplogroups. However, the haplogroups with the highest frequencies were most probably related to the mainstream of the population migration event. Other haplogroups with different genetic structure might have not involved in the same migration, e.g., came from other neighboring population groups in much recent time.

Haplogroups C and D were less frequent in the region, but both were found in MK and HM groups. The TK abundant haplogroup O1a2-M110 was observed only in one sample of Palyu which is known to have been genetically influenced by Kadai populations [31]. Haplogroups P and Q which predominate in North Asian populations were observed in MK and HM samples at low frequency (from 1.96% to 3.57%).

### Population comparison by clustering analyses

**Figure 4:**
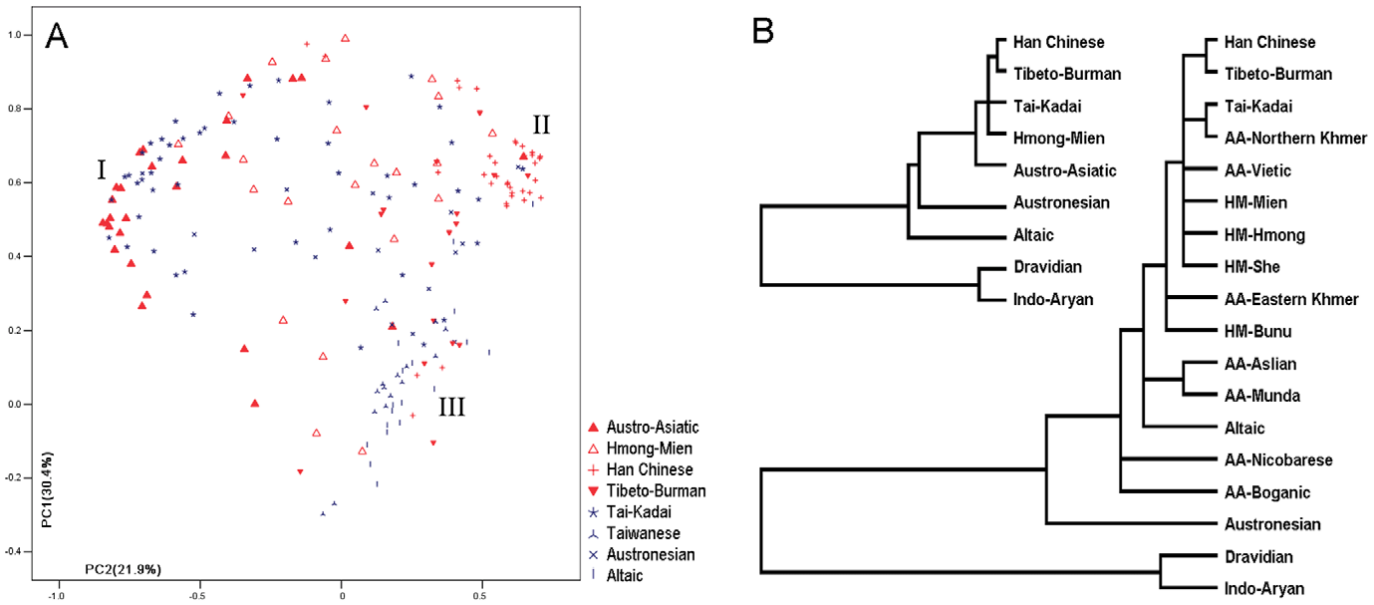
Clustering analyses for populations based on the Y chromosome SNP data. A: Principal component plot; B: Neighbor-joining tree.

In order to illustrate the close relationships between these two populations, HM and MK, and the distances to the other populations in East Asia, we collected the published haplotype frequency data from six other ethnic phyla [5], [6], [31] and applied principal component (PC) analysis. MK was included in the Austro-Asiatic super phylum together with the Munda subgroup. In the PC plot of Figure 4A, it is apparent that populations of Southeast Asia (Cluster I: Austro-Asiatic and Tai-Kadai) and East Asia (Clusters II and III) polarized in PC2. In PC1, the East Asian populations separated into two groups: coastal (Cluster III) and inland (Cluster II) groups. Interestingly, Hmong-Mien and Austronesian populations are scattered between the Southeast Asian and East Asian clusters. Therefore, HM group might be one of the intermediary groups from Southeast Asians to East Asians, illustrating the human migration from Southeast Asia into East Asia.

Furthermore, we also performed the hierarchical cluster analysis. Additional data of Indians (Dravidians and Indo-Aryans) were added as an outer group. Figure 4B contains two dendrograms of the clustering: populations in the same ethnic phylum were pooled in the upper dendrogram, while the subgroups of HM and Austro-Asiatic were distinguished in the lower one. In both of the dendrograms, the Han Chinese and the Tibeto-Burman are most closely clustered, with the Indians in the outer clade, which corresponds with the ethnic classification. With interlaced subgroups, the Austro-Asiatic and Hmong-Mien groups are rather difficult to distinguish.

### Network analyses of Major haplogroups

The STR diversity of a certain haplogroup is usually informative for the origin and diffusion of the relevant genetic markers. We analyzed STRs of the Y chromosomes belonging to O2a-M95, O3a3b-M7, and O3a3c1-M117 (see Table S1) to further delineate the relationship between MK and HM populations. In addition to the data from this study, we also included data from literature [4], [8], [18], [28] in subsequent analyses. The networks of these haplogroups based on STRs are presented in Fig. 2B. Outgroups (the STR data from upstream haplogroups of O2a-M95, O3a3b-M7, or O3a3c1-M117) were added to infer the origin of the network.

A clear hierarchical structure (annual ring shape) emerged in the network of O3a3b-M7 (Fig. 2B), in which MK haplotypes lay at the center of the network (immediately next to the origin), HM haplotypes were distributed at the periphery to the MK haplotypes, and the ST (here the subfamily Tibeto-Burman) haplotypes were only found further away from the origin. This hierarchical structure indicates bottleneck effects during the migration of O3a3b-M7 individuals from MK to HM and ST, with old haplotypes lost after population went through bottlenecks. The frequency of O3a3b is quite low in TK populations, and these individuals appeared sporadically in the network, sharing haplotypes with MK and HM. As the TK ethnic groups are located adjacent to MK and HM populations, the recent gene flow amongst the populations might have carried the O3a3b into TK populations.

In the O2a-M95 STR network (Fig. 2B), most of the big size haplotypes are shared by various ethnic groups, and no hierarchical expansion structure like that of O3a3b-M7 can be observed. Interestingly, the origin of the network is not in the center, indicating that the expansion of this haplogroup might have happened long time after its emergence. However, as we did not see any ethnic associated structure in the network, the expansion might have happened before the ethnic diversification in Southeast Asia. Therefore, the STR diversity of this haplogroup is not informative for the study of the northward migration and ethnic diversification of the early East Asians.

O3a3c1-M117 is another sub-haplogroup of O3-M122 and is much younger than M95. In the STR network of O3a3c1(Fig. 2B), more than half of the haplotypes can only be found in Sino-Tibetan populations, indicating the expansion of this haplogroup happened in the Sino-Tibetan populations. However, as the frequency of O3a3c1 might be higher than that of O3a3b in the earliest East Asians, the old haplotypes were not always lost along the way of northward migration through the bottlenecks which was not quite narrow. On another hand, the novel haplotypes in the periphery of the network are mostly in Sino-Tibetan populations while Hmong-Mien and Mon-Khmer were much closer to the origin, thus displaying the similar hierarchical structure as haplogroup O3a3b-M7.

Geographically, MK, HM, and ST populations are distributed across the border of Southeast Asia and East Asia in sequence from south to north. Thus, to examine the correlation between sample locations and STR haplotype structure of O3a3b, we plotted the latitude of each sample location against the STR mutation step number of each haplotype from the origin of the network (Fig. 3). Interestingly, the MK haplotypes were closest to the origin of the network genetically and are located in the southernmost area geographically, suggesting the migration of O3a3b started in the ancestors of MK region from the south. The HM haplotypes were further away (indicated by more mutation steps) from the network origin, and are located north of MK populations geographically. The ST haplotypes, which are most distant to the origin genetically, are located further north to those of HM. The correlation between latitude and mutation step was significant (r = 0.551, P = 2.18×10^−17^). Therefore, we propose that the O3a3b might have originated in the ancestors of MK populations, and they migrated northward unidirectionally into the ancestors of HM and ST populations sequentially. Moreover, a similar case happens to O3a3c1-M117 (Fig. 3, r = 0.442, P = 4.07×10^−14^), supporting the unidirectional northward migration suggested by the analyses on O3a3b.

However, we failed to construct this kind of annual ring shaped network from other haplogroups (Figure S1), because not all of the haplogroups were carried by the same migration through the same route.

### Time estimation for major haplogroups

There are several statistics for genetic time estimation. Here we calculated the ages of the O2a, O3a3b and O3a3c1 using the ρ statistic and average squared difference (ASD) which are suitable for the Y chromosome STR data (Table 2). The age of O3a3b is approximately 15295±2478 years by ρ statistic and 18804±4239 years by ASD. Both of these estimations fell into the Last Glacial Maximum of the Würm glacial age (20∼15 thousand YBP). That is also the time in which all of the ethnic specific (especially HM) sub-haplogroups of mtDNA emerged [32]. This same point has been made by other researchers [33].

**Table 2:** Time estimations for the three haplogroups (years).

Haplogroup	ρ Statistic^a^		ASD time^b^
	Age	Standard deviation	
O3a3c1-M117	24352	7325	18786±4565
O2a-M95	44376	10725	23913±3569
O3a3b-M7	15295	2478	18804±4239

a ρ Statistic was described in Forster et al [20].

b ASD time was described in Goldstein et al [22] and Stumpf et al [23].

The age of O3a3c1 is 24352±7325 years by ρ statistic and 18786±4565 years by ASD, a little bit older than that of O3a3b, just before the Last Glacial Maximum when early people migrated into East Asia through the Yun-Gui Plateau. Little diversity had been accumulated during the short time between its emergence and the start of migration. The unidirectional passage therefore has also left an effect on the STR structure of O3a3c1 although not as perspicuous as that of O3a3b. The oldest haplogroup O2a occurred thousands of years before the migration, and too much diversity had been accumulated to show any signals of the bottlenecks into East Asia. The subsequent ethnic diversification distributed most of the O2a STR haplotypes into different ethnic groups.

We had also implemented BATWING calculation. However, the result showed a much broader confidence interval (age of M7, 17–171 thousand years), and is not coincident with the known history of modern human. Therefore, we omitted the BATWING results.

## Discussion

### A Mon-Khmer origin of Hmong-Mien populations

The two population groups studied in this paper, i.e., the MK and HM, are indigenous populations of mainland Southeast Asia and Southwest China, respectively. This study has shown that MK and HM groups are closely related genetically and share high frequencies of haplogroups O2a-M95, O3a3b-M7, and O3a3c1-M117. The O3a3b-M7 is rare in neighboring populations, i.e. Tibeto-Burman and TK, while almost absent in the other East/Southeast Asian populations. The STR network of O3a3b-M7 (Fig. 2B) exhibits an obvious annual ring shape with no apparent gene flow from HM to MK but from MK to HM, suggesting that O3a3b-M7 in HM may have derived from those in MK, and those in ST subsequently derived from HM. O3a3c1-M117 might have the same history, exhibiting the similar hierarchical structure from MK to HM and ST (Fig. 2B). This finding is consistent with linguistic observation in which HM and Austro-Asiatic (including MK) linguistic groups are considered similar and could be classified into a super family named Proto-Yangtzean [34], [35].

The annual ring shape of the network or tree is not strange in human evolutionary studies. If we transform the Y haplogroup tree [16] of the world population into a circle, we will also see a similar annual ring shape with the Africans in the center while the Europeans and Asians in the periphery, and the Americans are even peripheral. It is widely accepted that this structure resulted from the bottlenecks between the continents through which early human populations have gone. The different between the structure of the world tree and our East Asian networks is that only two clades (CF and DE) touched out of the African cluster in the world tree while more clades were observed in the East Asian networks. As similar hierarchical structures were observed, and the most possible explanation can also be the bottleneck effects during the early human population migrated from Southeast Asia to East Asia.

### Genetic drift through a northward passage at the entrance of East Asians

Several studies have revealed that the genetic diversity is much higher in the southern populations than in the northern populations of East Asia, as the haplogroups found in the south include almost all the haplogroups in the north [5]. This pattern suggests that modern humans entered East Asia from the south and that Southeast Asia could have been a stopping point for migrants from Africa to East Asia. Thus, the unidirectional passage described in this paper could provide a more detailed account of the origin of East Asians especially those carrying O3a3b-M7 and O3a3c1-M117.

Unlike previous studies on the peopling of East Asia which focused on Y-SNP frequency [4], [5], we analyzed STR diversities of the haplogroups (Fig. 3). The positive correlation between latitudes of sample locations and STR mutational steps shows that populations in the north have younger STR haplotypes within the O3a3b and O3a3c1 haplogroups. When the networks were superimposed onto the maps (Information S1 and Information S2), that the view that O3a3b and O3a3c1 might have originated in the ancestors of MK populations and flowed into those of HM and ST populations became quite evident, and suggested a unidirectional diffusion of the relevant ancestral populations.

In an effort to interpret these findings, we need to take into account the topographic conditions at the time modern human arrived in Southeast Asia. In this regard, there were several geographic barriers to the distribution of human populations in Southeast Asia and South China, and these may have shaped the subsequent ethnic diversifications. For instance, the Annamese Cordillera segregated the ancestors of TK and MK in the Ice Age [36]. In our case, the jungles and mountains on the Yun-Gui Plateau were the buffer zone between the ancestors of HM and MK. The early population began to move through this large area of jungles and valleys from the Indo-China Peninsula around 19 thousand years ago, according to our estimate, around the Last Glacial Maximum [37]. The segregation effect of the mountains (Wuliang Mountains., Ailao Mountains., etc.) at that time was even stronger than today because of the cold climate. Several jokuls (Hengduan Mountains) on the north edge of the Yun-Gui Plateau formed impassable barriers [38]. Therefore, many “bottlenecks” occurred in the juncture region of Southeast Asia and East Asia, and only very small population could have gone along the Salween River or Mekong River through the Yun-Gui Plateau at that time, causing strong effect of genetic drift, which was indicated by the annual ring shaped networks of some Y chromosome haplogroups.

Judging from the structure of the O3a3b and O3a3c1 STR networks, the ancestral population size was changing during this migration but growing eventually, e.g. decreased through the bottlenecks and increased after the bottlenecks, enabling the rising of different diversity patterns. Moreover, the diffusion was very slow and dispersive because of the cold climate and complicated landforms through which it had to move. Consequently, genetic drift frequently occurred when populations moved from one valley to another, resulting in the loss of the old haplotypes and the emergence of the novel haplotypes in the new populations. Therefore, the STR haplotypes found at the forefront of the migrating population(s) would have gone through more steps averagely from the root of the network. It is likely that the STR diversity of O3a3b and O3a3c1 dispersed northward along a unidirectional passage, allowing more new haplotypes to evolve and move ahead eventually into new regions. This passage may extend through to North China, the homeland of modern Chinese and Tibetan, and O3a3b was almost lost during the long distance migration of those northern ST populations while O3a3c1 was increased by chance. This unidirectional passage is the combination of many collateral bottleneck effects, which resulted in the unique genetic structure and physical characteristics of East Asians and caused the genetic differences between East Asians and Southeast Asians [39]. The frequency of haplogroup O2a* is also high in our population samples, however, the old haplogroup O2a* did not exhibit similar diversity pattern as O3a3b because of its large “effective population size”.

At the same time, the unidirectional MK-HM-ST route through the Yun-Gui Plateau might not be the only route by which early people entered East Asia. The ancestors of TK and Austronesians might have entered East Asia through a different coastal route [28]. Some populations might have also entered East Asia from the northwest much recently [40]. Thus, studies of more populations using large sets of informative markers will certainly provide a more detailed picture of the origins of East Asian populations.

## Supporting Information

Protocol S1 **The primers and protocols for the SNPs and STRs genotyping used in this paper.**
(DOC)

Figure S1 **STR networks for the minor haplogroups found in MK and HM.**
(TIF)

Table S1 **Y chromosome STR data of the MK and HM samples and topology showing the hierarchy between SNPs.** Note: Y-STR data of following populations are unavailable due to poor sample quality or lack of data from references: Pahng, Northern Bunu, Mien Western, Guizhou Miao, Yunnan Miao, Blang, Ava, Kinh, Muong. Hence only 1360 sets of Y-STR data were listed.
(XLS)

Information S1 **The action map reproducing a unidirectional diffusion northward from the population of MK to HM and ST according to the individual STR mutations of O3a3b-M7 and latitude of each population.**
(GIF)

Information S2 **The action map reproducing a unidirectional diffusion northward from the population of MK to HM and ST according to the individual STR mutations of O3a3c1-M117 and latitude of each population.**
(GIF)
