# Supplementary material for: Human Migration through Bottlenecks from Southeast Asia into East Asia during Last Glacial Maximum Revealed by Y Chromosomes
Source: PLoS One. 2011 Aug 31;6(8):e24282. doi: 10.1371/journal.pone.0024282 (PMC3164178; doi:10.1371/journal.pone.0024282)
Supplement: Protocol S1 — The primers and protocols for the SNPs and STRs genotyping used in this paper. (DOC) [file pone.0024282.s001.doc]

**Y chromosome PCR with fluorescent primers**

100mM dNTPs(dATP,dGTP,dTTP,dCTP) were purchased from Shanghai Promega. HotStarTag DNA Polymerase, 10﹡Reaction Buffer, 25mM MgCl_2_ and 5﹡Q solution were purchased from QIAGEN.

M121-F：FAM-5' ACA AAG ACC TGG ACA GAT TAC 3'

M121-R：5' CCC TTA AAA ACA GCA TGA TA 3'

PCR product length: 123bp, del-118bp

M117-F-N：FAM-5' GTA CGA AGA AAA TCA AGG CTA TTA 3'

M117-R-N：5' TTG GGT AGA AAA ACT GCA AGT AG 3'

PCR product length: 317bp, del-313bp

M175-F-N：FAM-5' TTG AGC AAG AAA AAT AGT ACC CA 3'

M175-R-N：5' TTC AGT TAG CCT TGA TTG ACT GT 3'

PCR product length: 226bp, del-221bp

M134-F-N：5' AGA ATC ATC AAA CCC AGA AGG 3'

M134-R-N：NED(or HEX) -5' TCT TTG GCT TCT CTT TGA ACA G 3'

PCR product length: 232bp, del-231bp

M15F：FAM-5’-ACA AAT CCT GAA CAA TCG C-3’

M15R：5’-GTC TGG GAA GAG TAG AGA AAA G-3’

PCR product length: 142bp, ins-151bp

**Cycle condition:**

94℃﹡15min - [94℃﹡30sec - 65℃(-0.5℃/cycle)﹡90sec - 72℃﹡1min]﹡20cycles – [89℃﹡30sec - 55℃﹡90sec - 72℃﹡1min]﹡15cycles - 60℃﹡30min - 4℃﹡∞

**PCR-RFLP typing protocol**

Restriction enzymes were purchased from New England BioLabs; 100mM dNTPs(dATP,dGTP,dTTP,dCTP), Tag DNA Polymerase, 10﹡Reaction Buffer, 25mM MgCl_2_ were purchased from Shanghai Promega.

**YAP**

**Primers:**

YAP-F: 5'CAG GGG AAG ATA AAG AAA TA 3'

YAP-R: 5'ACT GCT AAA AGG GGA TGG AT 3'

**Cycle condition:**

94℃﹡3min - [94℃﹡20sec - 51℃﹡40sec - 72℃﹡40sec]﹡34 cycles - 72℃﹡5min - 4℃﹡∞

**Allele calling:** YAP-(150bp), YAP+(400bp)

**M130(C to T mutation at position 41)**

**Primers:**

M130-RFLP-F: 5’ TAT CTC CTC TTC TAT TGC AG 3’

M130-RFLP-R: 5’ CCA CAA GGG GGA AAA AAC AC 3’

**Cycle condition:**

94℃﹡3min - [94℃﹡20sec - 55℃﹡30sec - 72℃﹡30sec]﹡34 cycles - 72℃﹡5min - 4℃﹡∞

**PCR product:** 205bp

**Enzyme:** BslI (CCNNNNN/NNGG)

**Allele calling:** C-Cutting(162bp + 43bp),T-NO Cutting(205bp)

**M89(C to T mutation at position 347)**

**Primers:**

M89-RFLP-F: 5’ GAA AGT GGG GCC CAC AGA AGG A 3’

M89-RFLP-R: 5’ GCA AAT CAG GCA AAG TGA GAC AT 3’

**Cycle condition:**

94℃﹡3min - [94℃﹡25sec - 53℃﹡25sec - 72℃﹡30sec]﹡34 cycles - 72℃﹡5min - 4℃﹡∞

**PCR product:** 100bp

**Enzyme:** NlaⅢ(CATG)

**Allele calling:** C-Cutting(77bp + 23bp),T-NO Cutting(100bp)

**M9(C to G mutation at position 68)**

**Primers:**

M9-RFLP-F: 5’GAA ACG GCC TAA GAT GGT TGG AT 3’

M9-RFLP-R: 5’AAA CTG AAT CTT TTT TCC TCA TTT TTG 3’

**Cycle condition:**

94℃﹡3min - [94℃﹡25sec - 55℃﹡25sec - 72℃﹡30sec]﹡34 cycles - 72℃﹡5min - 4℃﹡∞

**PCR product:** 210bp

**Enzyme:** BamHI(GGATCC)

**Allele calling:** C-Cutting(190bp + 20bp),G-NO Cutting(210bp)

**M122(T to C mutation at position 73)**

**Primers:**

M122-RFLP-F: 5’ TAG AAA AGC AAT TGA GAT ACT AAT TCA 3’

M122-RFLP-R: 5’ GCG ATG CTG ATA TGC TAG TTC AG 3’

**Cycle condition:**

95℃﹡3min - [94℃﹡20sec - 65℃(-0.5℃/cycle)﹡25sec - 72℃﹡30sec]﹡14cycles – [94℃﹡20sec - 55℃﹡25sec - 72℃﹡20sec]﹡20 cycles - 72℃﹡5min - 4℃﹡∞

**PCR product:** 122bp

**Enzyme:** NlaⅢ(CATG)

**Allele calling:** T-Cutting(100bp + 22bp),C-NO Cutting(122bp)

**M7(C to G mutation at position 216)
Primers:**

M7-RFLP-F:5'TGT ACC CTT GAC CAA TGC CTT 3' M7-RFLP-R:5'TTG TAG TTG AGT TAC TGT TCT TCT A 3'

**Cycle condition:**

95℃﹡3min - [94℃﹡20sec - 65℃(-0.5℃/cycle)﹡25sec - 72℃﹡30sec]﹡14cycles – [94℃﹡20sec - 55℃﹡25sec - 72℃﹡20sec]﹡20 cycles - 72℃﹡5min - 4℃﹡∞

**PCR product:** 126bp(PCR product should be purified before digestion)

**Enzyme:** BfaI(CTAG)

**Allele calling:** G-NO Cutting(126bp),C-Cutting(100bp + 26bp)

**M164( T to C mutation at position 329)**

**Primers:**

M164-RFLP-F:5' GTGCCAGGCATCAAGCAGC 3'
M164-RFLP-R:5'GGACAATTTCTTCTCCTTGTGA 3'

**Cycle condition:**

95℃﹡3min - [94℃﹡20sec - 65℃(-0.5℃/cycle)﹡25sec - 72℃﹡30sec]﹡14cycles – [94℃﹡20sec - 55℃﹡25sec - 72℃﹡20sec]﹡20 cycles - 72℃﹡5min - 4℃﹡∞

**PCR product:** 129bp

**Enzyme:** AluI(AGCT)

**Allele calling:** T-Cutting(111bp+18bp),C-NO Cutting(129bp)

**M159 (A to C mutation at position 89)**

**Primers:**

M159-RFLP-F: 5' ATTGGATTGATTTCAGCCTTC 3'

M159-RFLP-R: 5' CTC TGG AGT CGA AAG AGCG 3'

**Cycle condition:**

95℃﹡3min - [94℃﹡20sec - 65℃(-0.5℃/cycle)﹡25sec - 72℃﹡30sec]﹡14cycles – [94℃﹡20sec - 55℃﹡25sec - 72℃﹡20sec]﹡20 cycles - 72℃﹡5min - 4℃﹡∞

**PCR product:** 108bp

**Enzyme:** BsrBI(CCGCTC)

**Allele calling:** A-NO Cutting(108bp), C-Cutting(91bp+17bp)

**M119(A to C mutation at position 224)**

**Primers:**

M119-RFLP-F : 5’ AGG TAA ATG ACT CAC CCT AAG GAA G 3’

M119-RFLP-R: 5’ GGG TTA TTC CAA TTC AGC ATA CAC GC 3’

**Cycle condition:**

94℃﹡3min - [94℃﹡25sec - 56℃﹡30sec - 72℃﹡30sec]﹡34 cycles - 72℃﹡5min - 4℃﹡∞

**PCR product:** 161bp

**Enzyme:** BstuI(CGCG)

**Allele calling:** C-cutting(135bp+26bp), A-NO cutting(161bp)

**M101( C to T mutation at position 154)**

**Primers:**

M101-RFLP-F: 5' TTTAGCCAAAATATTTTTTTTGTA  3'
M101-RFLP-R: 5' GCTTACTCTTGCTGAACCCTA  3'

**Cycle condition:**

95℃﹡3min - [94℃﹡20sec - 65℃(-0.5℃/cycle)﹡25sec - 72℃﹡30sec]﹡14cycles – [94℃﹡20sec - 55℃﹡25sec - 72℃﹡20sec]﹡20 cycles - 72℃﹡5min - 4℃﹡∞

**PCR product:** 139bp

**Enzyme:** RsaI(GTAC)

**Allele calling:** C-Cutting(116bp+23bp),T-NO Cutting(139bp)

**M110(T to C mutation at position 241)**
**Primers:**

M110-RFLP-F: 5'AAC ATT CTC TGT AGA CTC ACT GG3'
M110-RFLP-R: 5'ATT TAG CAC TTC TTT TCC CC3'
**Cycle condition:**

95℃﹡3min - [94℃﹡20sec - 65℃(-0.5℃/cycle)﹡25sec - 72℃﹡30sec]﹡14cycles – [94℃﹡20sec - 55℃﹡25sec - 72℃﹡20sec]﹡20 cycles - 72℃﹡5min - 4℃﹡∞

**PCR product:** 200bp

**Enzyme:** NlaⅢ(CATG)

**Allele calling:** T-NO Cutting(200bp),C-Cutting(108bp+92bp)

**M95( C to T mutation at position 172)
Primers:**

M95-RFLP-F: 5’ ATA AGG AAA GAC TAC CAT ATT AGC G 3’

M95-RFLP-R: 5’ TTT GAA GGC CCC AGT TGT GAG 3’
**Cycle condition:**

95℃﹡3min - [94℃﹡20sec - 59℃(-0.5℃/cycle)﹡25sec - 72℃﹡30sec]﹡14cycles – [94℃﹡20sec - 53℃﹡25sec - 72℃﹡20sec]﹡20 cycles - 72℃﹡5min - 4℃﹡∞

**PCR product:** 202bp

**Enzyme:** HhaI(GCGC)

**Allele calling:** C-cutting(178bp+24bp), T-NO cutting(202bp)

**M88(A to G mutation at position 166)**

**Primers:**

M88-RFLP-F: 5’CTG TAG CCT AGA GCC TGC CAA A 3’

M88-RFLP-R: 5’TAG AGA GGA AAA CCT ATC TTG GAT G 3’

**Cycle condition:**

95℃﹡3min - [94℃﹡20sec - 65℃(-0.5℃/cycle)﹡25sec - 72℃﹡30sec]﹡14cycles – [94℃﹡20sec - 55℃﹡25sec - 72℃﹡20sec]﹡20 cycles - 72℃﹡5min - 4℃﹡∞

**PCR product:** 161bp

**Enzyme:** HhaI(GCGC)

**Allele calling:** A-NO cutting(161bp),G-cutting(140bp+21bp)

**M120 (T to C mutation at position 224)**

**Primers:**

M120-RFLP-F: 5’ TGG ACA GAT TAC AGT AAA CCT TCA AC 3’

M120-RFLP-R: 5’ GTA TAA TTT CCC TTA AAA ACA TCA TG 3’

**Cycle condition:**

95℃﹡3min - [94℃﹡20sec - 65℃(-0.5℃/cycle)﹡25sec - 72℃﹡30sec]﹡14cycles – [94℃﹡20sec - 55℃﹡25sec - 72℃﹡20sec]﹡20 cycles - 72℃﹡5min - 4℃﹡∞

**PCR product:** 123bp

**Enzyme:** BspHI(TCATGA)

**Allele calling:**T-cutting (100bp+23bp), C-No cutting (123bp)

**M45(G to A mutation at position 109)**

**Primers:**

M45-RFLP-F: 5’ATT GGC AGT GAA AAA TTA TAG CTA 3’

M45-RFLP-R: 5’TGC CTT TGC TAC AAC TCT CCT A 3’

**Cycle condition:**

94℃﹡3min - [94℃﹡25sec - 55℃﹡30sec - 72℃﹡30sec]﹡34 cycles - 72℃﹡5min - 4℃﹡∞

**PCR product:** 162bp

**Enzyme:** BfaI(CTAG)

**Allele calling:** G-cutting(140+22bp), A-NO cutting(162bp)

**D3-P47** was typed using a PCR-RFLP assay according to Gayden et al. 2007.

**D-M174, D2-M55, NO-M214, N1-LLY22g, N1a-M128, N1b-P43, N1c-M46, P31-O2, O3a-M324** were typed using a TaqMan® SNP genotyping assay.

**Y-STR**

We used AmpFLSTR®YfilerTM PCR Amplification Kit from Appliedbiosystems to conduct the PCR.

**Cycle condition:**

95℃﹡11min - [94℃﹡1min - 61℃﹡1min - 72℃﹡1min]﹡30 cycles - 72℃﹡1min- 60℃﹡80min - 4℃﹡∞
