# Supplementary figures and images for: Human Migration through Bottlenecks from Southeast Asia into East Asia during Last Glacial Maximum Revealed by Y Chromosomes
Source: PLoS One. 2011 Aug 31;6(8):e24282. doi: 10.1371/journal.pone.0024282 (PMC3164178; doi:10.1371/journal.pone.0024282)

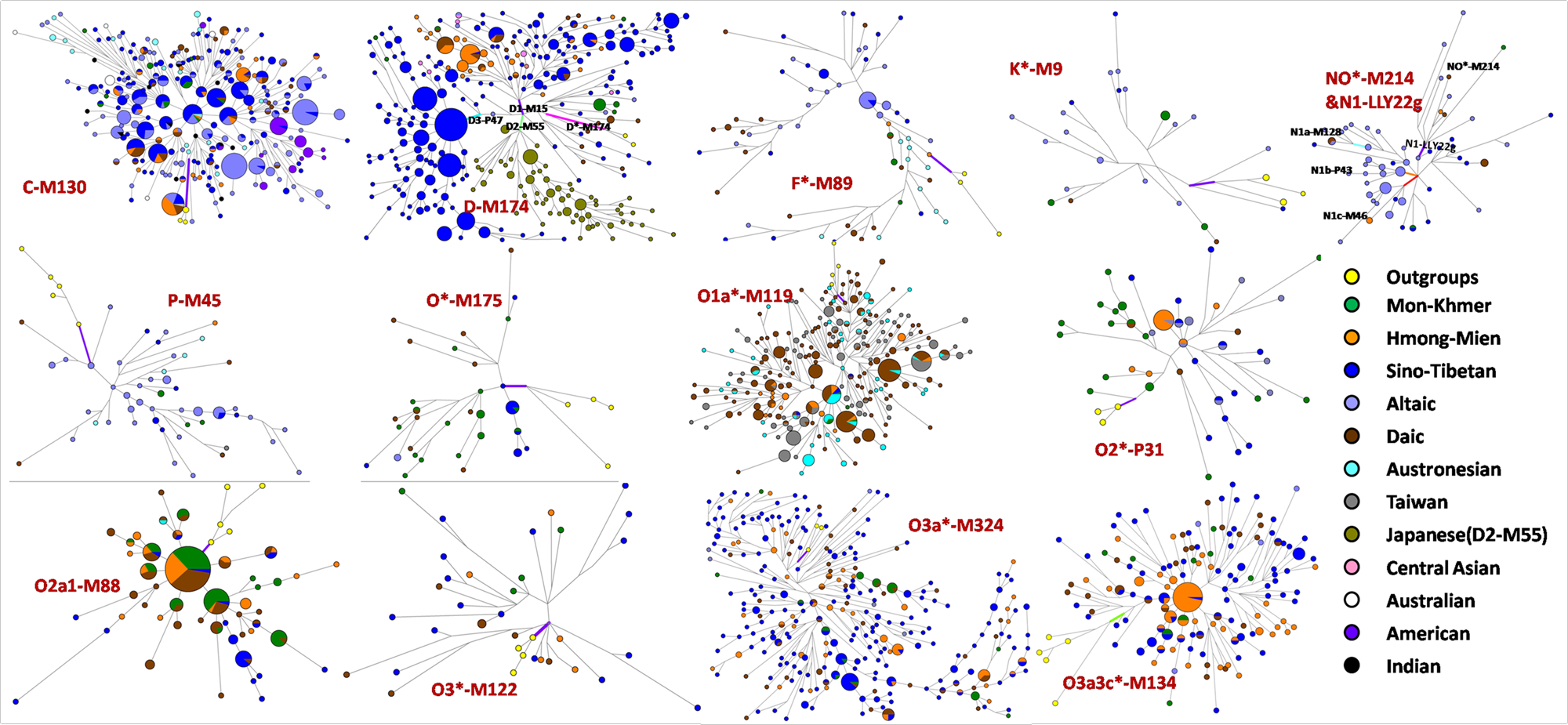

Supplement: Figure S1 — STR networks for the minor haplogroups found in MK and HM. (TIF) [file pone.0024282.s002.tif]

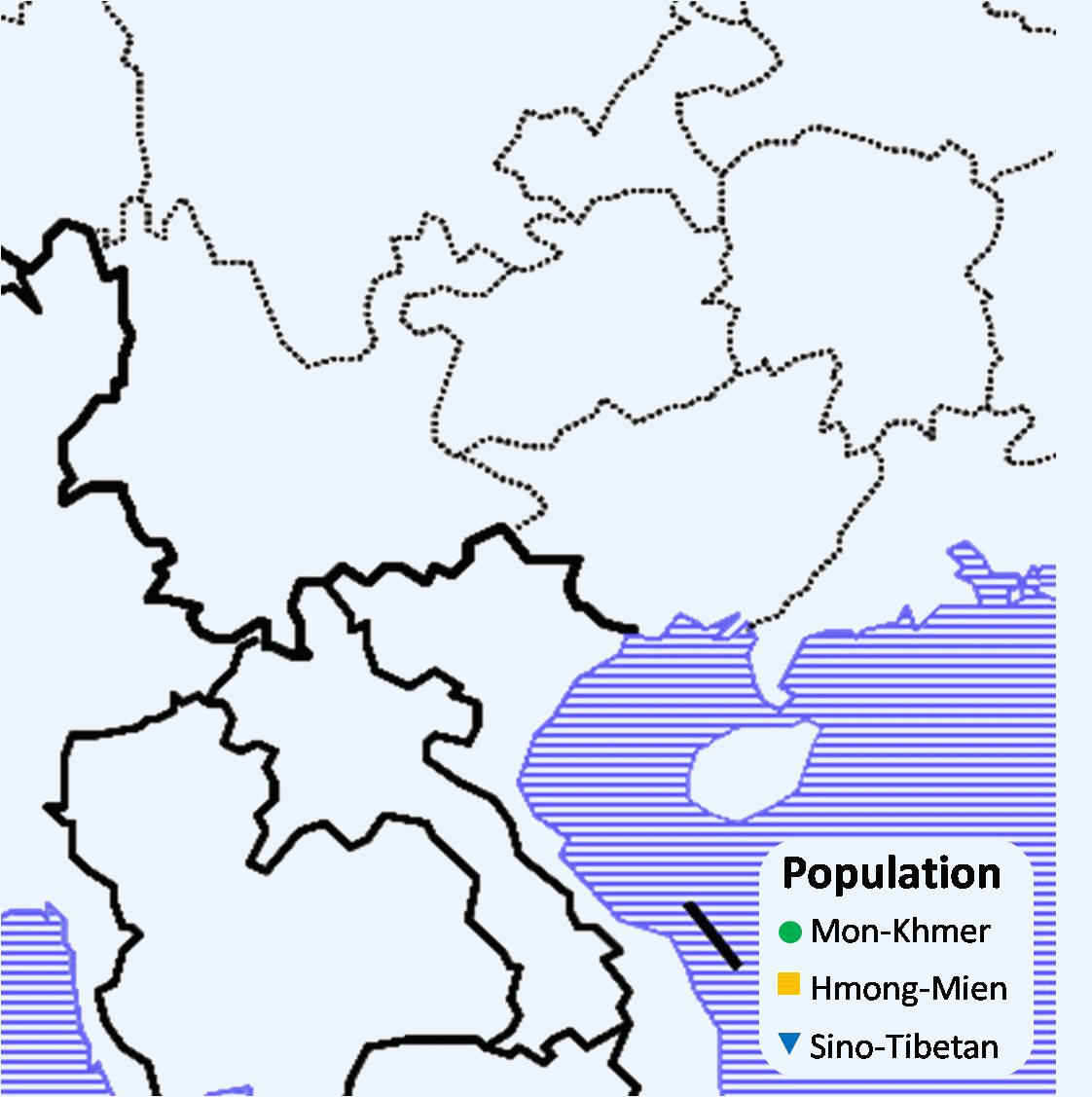

Supplement: Information S1 — The action map reproducing a unidirectional diffusion northward from the population of MK to HM and ST according to the individual STR mutations of O3a3b-M7 and latitude of each population. (GIF) [file pone.0024282.s004.gif]

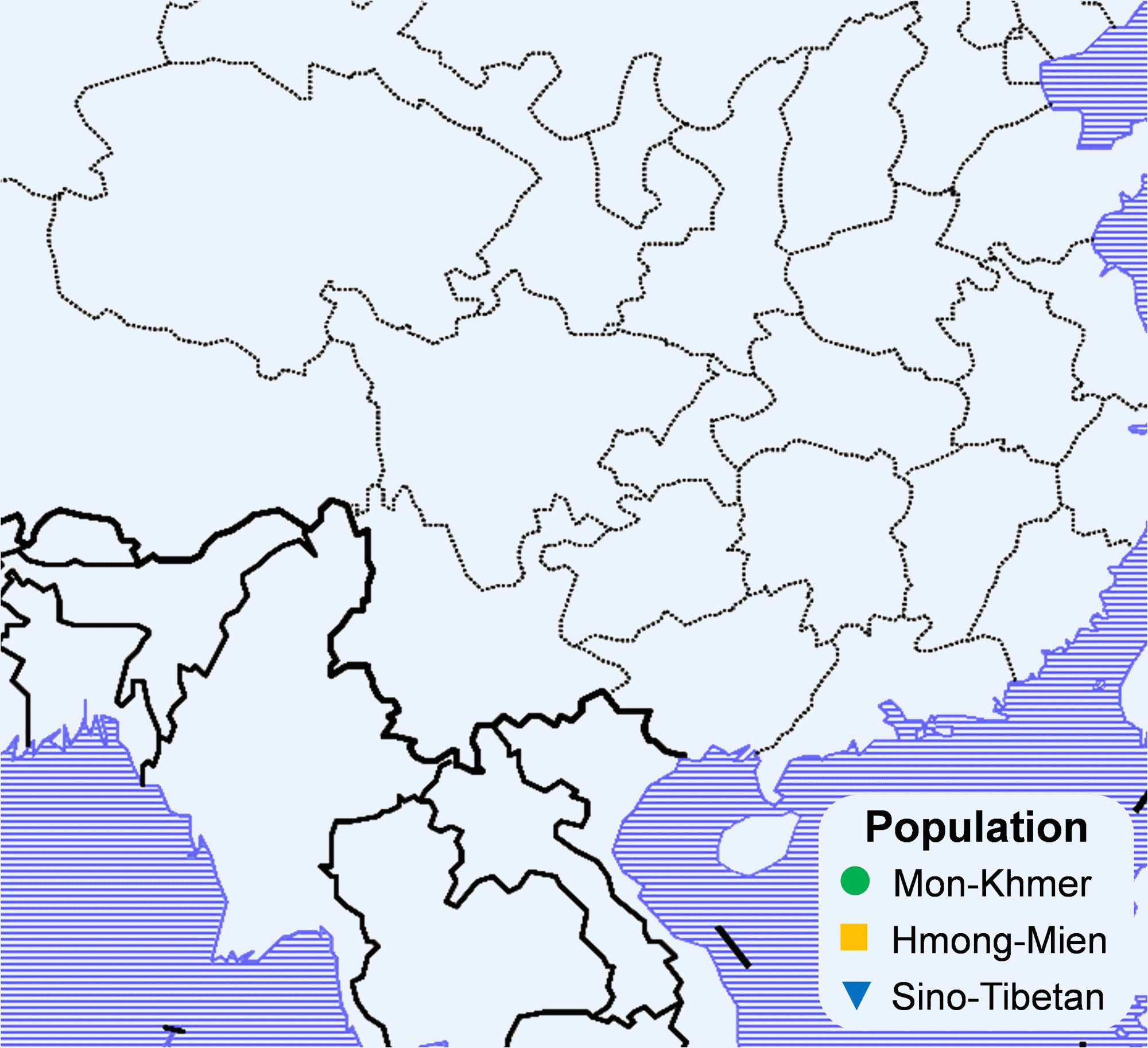

Supplement: Information S2 — The action map reproducing a unidirectional diffusion northward from the population of MK to HM and ST according to the individual STR mutations of O3a3c1-M117 and latitude of each population. (GIF) [file pone.0024282.s005.gif]
